# A rare presentation of rhino-orbital-cutaneous mucormycosis in an immunocompetent patient: a case report

**DOI:** 10.11604/pamj.2024.48.13.43454

**Published:** 2024-05-15

**Authors:** Ni Putu Ayu Reza Dhiyantari, Delfitri Lutfi, Dwi Hari Susilo, Irwan Kristyono, Alicia Widya

**Affiliations:** 1 Department of Ophthalmology, Faculty of Medicine Airlangga University, Dr Soetomo Hospital Surabaya, Surabaya, Indonesia,; 2 Department of Head and Neck Surgery, Faculty of Medicine Airlangga University, Dr Soetomo Hospital Surabaya, Surabaya, Indonesia,; 3 Department of Ear, Nose and Throat (ENT), Faculty of Medicine Airlangga University, Dr Soetomo Hospital Surabaya, Surabaya, Indonesia,; 4 Department of Microbiology, Faculty of Medicine Airlangga University, Dr Soetomo Hospital Surabaya, Surabaya, Indonesia

**Keywords:** Mucormycosis, antifungal, fungal infection, communicable, case report

## Abstract

Mucormycosis is a rare opportunistic infection caused by Mucorales fungi. Cutaneous mucormycosis typically present as chronic indolent infection, whereas rhino-orbital mucormycosis is rapidly progressive disease often invade the adjacent cerebral tissue associated with high mortality. This case represents the atypical clinical history of rhino-orbital-cutaneous mucormycosis. The patient was presented with a right orbital cellulitis associated with an extensive multiple suppurative deep cutaneous infection and worsening headache. The skin lesion was initiated from a localized abscess at the right periorbital area nine months before admission. Suspicion of fungal infection was raised after weeks of non-responsive antibiotics treatment. Aggressive treatment with exoneration of the right eye and surgical debridement was undertaken. Periodic acid Schiff staining from healthy periorbital tissue revealed ribbon-like hyphae with pauciseptate and 90° branching identified as Mucoraceaefamily. The resolution was seen after four weeks of antifungal treatment with Amphotericin B.

## Introduction

Mucormycosis, also known as zygomycosis, refers to several different diseases caused by fungal infection belongs to the order of Mucorales. The specific fungal genus involved is usually Mucor or Rhizopus [1]. Mucormycosis infections are invasive and life-threatening [2]. Mucormycosis is a rare opportunistic infection, which represents the third most common Anglo-invasive fungal infection after candidiasis and aspergillosis. Early diagnosis and treatment are crucial due to high mortality and morbidity [3]. The incidence of mucormycosis is rising over several years, especially during coronavirus pandemic all around the globe. The incidence is notably very high in the Asian continent [3], especially In India, where the prevalence of mucormycosis is approximately 70 times higher than the worldwide-estimated rate [4]. The most common presentation was rhino-orbit-cerebral, followed by cutaneous, pulmonary, oral cavity involvement and gastrointestinal [5]. The rhino-orbital localization is usually manifested by a rapidly invasive acute rhino sinusitis with ophthalmological signs and symptoms. Successful mucormycosis treatment requires correction of the underlying risk factor(s), antifungal therapy, and aggressive debridement surgery [6]. We report the case of a rhino-orbital-cutaneous mucormycosis in an immunocompetent patient who presented a right orbital cellulitis associated with an extensive multiple suppurative deep cutaneous infection in a chronic, progressive manner. Suspicion of fungal infection was raised after weeks of inconclusive diagnostic modalities and unresponsive treatment.

## Patient and observation

**Patient information:** a 22-year-old male from rural East Java, came to the orbital oncology outpatient clinic at Dr Soetomo General Hospital with chief complaint of open wound at the inner corner of right eye and the right face, accompanied by protrusion of the right eye. Thick yellowish fluid secrets continuously oozing from the wound since 5 months before admission. The patient also complained of pain in the right periorbital area radiating to the right head since 2 weeks before admission. No history of juvenile or childhood diabetes mellitus, there were neither family history of diabetes nor immunodeficiency condition in the family. Patient denied history of traumatic event or accident. Patient previously treated with oral antibiotics from general practitioner without significant improvement.

**Figure 1:**
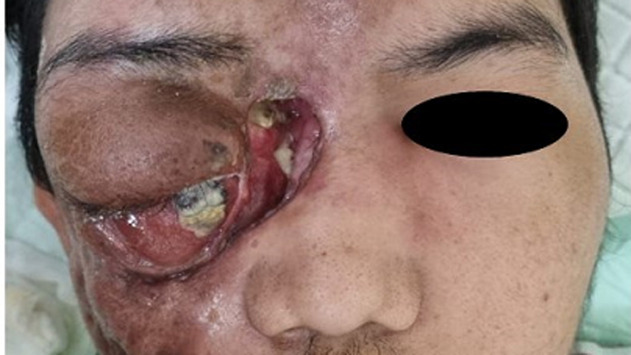
a-22-year-old male with two big ulcers at the right medial canthus and right periauricular area; multiple fistulas present at right zygomatic and maxillary area; severe proptosis with prolapse of ocular content was observed on the right eye

**Clinical findings:** the wound begins to develop 9 months before admission, it was initially only a small bump at the size of a corn kernel in the 1/3 medial of lower eyelid region. The bump increased in size and ruptured, releasing thick yellowish fluid mixed with blood. New bumps develop on multiple sites around right eye and right hemifacial. The right eye started to swell and blurry since 2 months before admission. He also complained of worsening pain originating from the back of the right eye, radiating to the right head for two weeks before admission. On admission, the right eye has no light perception and no ocular motility. Left eye visual acuity was 20/20, with normal ocular motility. Exophthalmos of the right eye could not be measured due to severe tissue edema and ulcer at the lateral canthus. From ophthalmologic examination, we found palpebra oedema at both eyes, with hyperemia and hyperpigmentation on the right eye. The content of the eyeball was already prolapsed (Figure 1). Patient was very poorly responded to antibiotic treatment. Patient was showing no clinical improvement and even worsening inflammation of frontal, left periorbital, and left zygomatic area after twelve days of Pennicillin G and Moxifloxacin. There was also enormous abscess formation on the frontal area and left zygomatic area, accompanied by swelling of the labial area.

**Figure 2:**
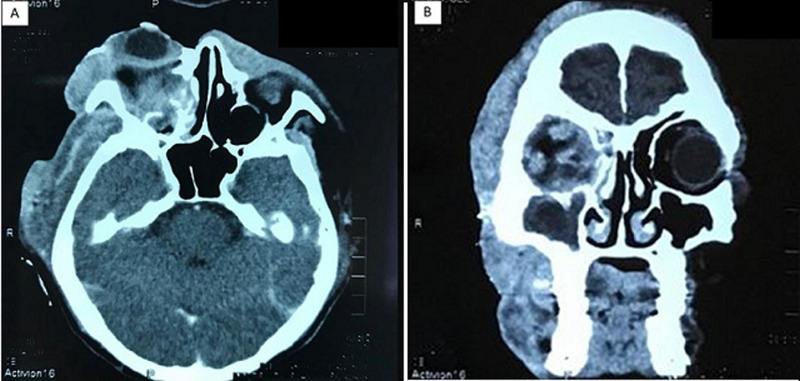
A) head CT scan with contrast, axial slice showed proptosis of the right eye 2.9 cm from interzygomatic line; solid mass was seen in the right retrobulbar, filling intraconal and extraconal area, with heterogenous contrast enhancement; this mass initially raising suspicion of malignancy; B) coronal slices showed thickening of the right extraocular muscles indicating chronic myositis

**Figure 3:**
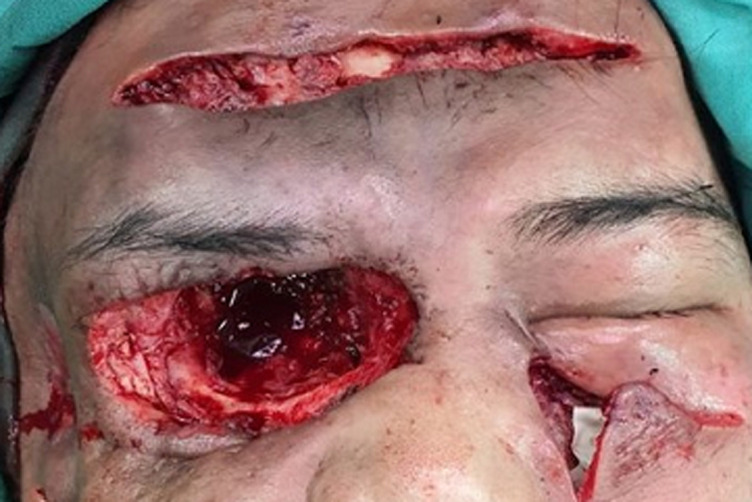
exenteration and surgical debridement of the frontal and zygoma subcutaneous abscess

**Figure 4:**
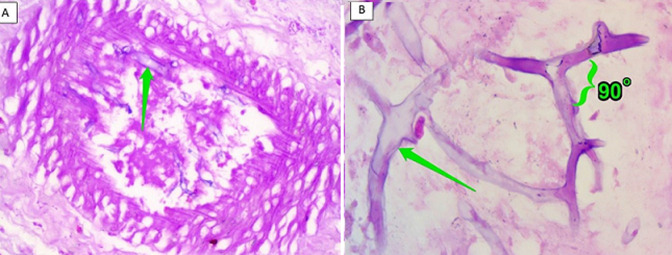
A) periodic acid Schiff staining revealed ribbon-like hyphae with pauciseptate (arrow); B) some of the hyphae with 90° branching identified as mucor sp (arrow)

**Diagnostic assessment:** ancillary testing to exclude immunocompromising conditions i.e. HIV infection, Hepatitis B and C, autoimmune disease screening, Tuberculosis GenXpert testing were unremarkable. Head CT-scan with contrast confirming orbital cellulitis and proptosis of the right ocular bulb approximately 2.9 cm to the inferolateral side with cutan-subcutaneous abscess formation (Figure 2). Multidisciplinary discussion conclude that it was most likely a fungal infection and that aggressive measures should be taken immediately. Debridement surgery with a Weber-Fergusson technique and exenteration of the right orbital tissue was carried out on day 20^th^ of admission (Figure 3). Fungal bodies were identified from histopathological slides of periorbital tissue biopsy. Periodic acid Schiff staining revealed ribbon-like hyphae with pauciseptate, some of the hyphae with 90° branching identified as mucoraceae family (Figure 4).

**Diagnosis:** rhino-orbital-cutaneous mucormycosis.

**Therapeutic interventions:** multidisciplinary discussion conclude that it was most likely a fungal infection and that aggressive measures should be taken immediately. Debridement surgery with a Weber-Fergusson technique and exenteration of the right orbital tissue was carried out on day 20^th^ of admission. Treatment was immediately switched to antifungal regiment following diagnostic establishment of mucormycosis. Amphotericin B 50 mg/day for 5 weeks was given during admission, with significant improvement. Itraconazole 200 mg q 8 hours continued for 6 months after patient was discharged from the hospital. Left eye was closely monitored.

**Figure 5:**
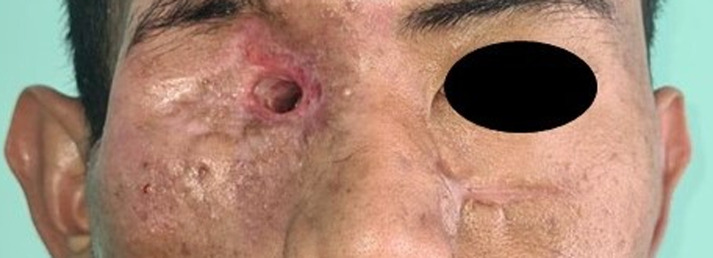
follow-up six months after treatment; the exenterated socket has epithelialized, but the fistula to the maxillary sinus is still present; an orbital prosthesis has been proposed and will be implemented in the near future

**Follow-up and outcome of interventions:** major clinical improvement was seen at fifth day post exenteration and surgical debridement and the third day antifungal administration, with decreasing pus production, alleviation of inflammation signs and symptoms, resolution of left preseptal cellulitis and healthy granulation tissue development over debrided wound and exenterated wound of the right eye. Patient was discharged five weeks after antifungal administration, with close follow-ups. No recurrence was found at six months follow up. Exenterated socket was epithelialized, but a fistula to maxillary sinus is still present (Figure 5)

**Patient perspective:** I was sad and angry at first when I found out that the disease had taken my right eye and I had to live with a deformity in my face, but now I have learned that the disease could have taken my life too if the surgery had not been done. I already accept this condition as Allah's will, and this is my second chance. Thank you to all the doctors and nurses who cared for me during my illness.

**Informed consent:** patient and family were acknowledged about the case reported and agreed that the case would be published for the benefit of people and medicine.

## Discussion

Mucormycosis is a fungal infection caused by fungi in the order of mucorales. Most cases of mucormycosis are caused by the genera rhizopus and mucor. Mucorales are ubiquitous fungi that are usually found in soil, compost, animal feces, decaying vegetables, agricultural debris, or other organic matter and in association with plants, fungi, animals, and humans as opportunistic pathogens [2]. Inhalation is the main port de entry responsible for rhino-orbital and pulmonary forms. Orbital mucor infection, almost always extend from adjacent sinus or the nasal cavity [1]. In several published cases of immunocompetent mucormycosis, the infection was localized to the skin following trauma [5]. Its high morbidity and mortality is associated to its rapid angio-invasion behavior and tissue necrosis [7]. In this case, the most probable port de entry is via a minor skin lesion started 9 months before admission. Cutaneous mucor infection developed slowly it eventually invades orbital structures, manifested as rhino-orbital-cutaneous form.

A recent study from India found that mucormycosis was predominant in males (69.6%) rather than females (19.6%), and most of the patients were active COVID-19 cases (70.5%) [8]. The most common affected age group was that between 31-60 years [3]. The case illustrated in this report represent an unusual course of illness, due to young age, no risk factors or immunocompromising conditions, and also the rhino-orbital-cutaneous form of the disease. Orbital mucormycosis typically presents with proptosis and an orbital apex syndrome, which manifested as internal and external ophthalmoplegia, ptosis, decreased corneal sensation, and decreased vision [1]. Facial and ocular warning symptoms including eyelid, periocular, and facial edema as well as discoloration, regional pain, proptosis, sudden loss of vision, facial paresthesia, sudden ptosis, ocular motility restriction and diplopia, and also facial palsy. Other symptoms that should raise the awareness of cerebral dissemination are worsening headache, fever, altered sensorium, paralysis and focal seizures [4]. The most common clinical feature in the case series was the presence of facial pain or headache. They suggest that facial pain or headache could be a hallmark symptom when faced with a patient with chronic paranasal sinus disease with evident computed tomograph (CT) scan sinus occupation [7].

Diagnosis establishment in this case alone was very challenging due to atypical presentation and rarity of the case reported in Indonesia. The orbital apex syndrome in the case presented was fully blown, but the only nasal symptom reported was nasal stuffiness. Multiple deep cutaneous abscess and fistula formation suggest the diagnosis of deep fungal infection. The suspicion of Mucor infection was made later at the third week of unresponsive antibiotic treatment. Proven mucormycosis infection established by mycological evidence of mucormycosis in tissue biopsy taken during sinus debridement or from the orbital biopsy [4]. Identification of mucoraceae fungi in this case was established from periodic acid Schiff staining of the histopathological slides that were taken from the first surgery. Ribbon-like hyphae with pauciseptate, some of the hyphae with 90° branching were characteristic of mucoraceae family. The treatment of mucormycosis is difficult due to multiple sites of infection in the host, various species causing the disease, and different duration required for therapy.

Characteristic angioinvasion from mucor infection results in hematogenous dissemination of the organism, whereas necrosis of the affected tissues prevents penetration of immune cells and antifungal agents to the infection. The effective approach for the treatment of mucormycosis usually based on the combination of Amphotericin B, timely surgical debridement of involved tissues (especially in sino-orbital and cutaneous forms), and finally control of underlying conditions [8]. Full-dose liposomal Amphotericin B is the drug of choice in the case of Mucor infection. The use of Amphotericin B Deoxycholate in patients with good renal function is acceptable where liposomal Amphotericin B is not available [9]. Amphotericine B intravenous (IV) infusion administration is given over 2 to 6 hours IV drip or pump [10]. About 80% of the patients will develop either infusion-related adverse effect or renal toxicity. Infusion related adverse effects are fever, hypertension, chills, or nausea. Premedication 30 to 60 minutes before administration with a combination of paracetamol plus diphenhydramine might be given to reduce infusion-related adverse effects [10].

At our center, liposomal Amphotericine B was not available. For that reason, Amphotericin B deoxycholate IV infusion was given for 5 weeks with close monitoring of the adverse effect. In addition, Itraconazole 200 mg q 8 hours was given orally and subsequently continued after the patient was discharged. Itraconazole is to be given for 6 months and slowly tapered off thereafter. The case illustrated present atypical clinical sign and symptoms of chronic mucormycosis infection in an otherwise healthy young man. Atypical clinical presentation leads to delayed diagnosis and severe disease progression. The management of this case was based on multidisciplinary approach, copious team discussion, and literature reviews. Further follow-up was continued at orbital-oncology outpatient clinic, dermato-venerology clinic, and plastic surgery outpatient clinic. Family member was trained to do wound care at home every two to three days. Reconstruction surgery with skin graft is to be performed in the future to close the major surgical defect over the frontal and right orbital area. Orbital prosthesis is to be proposed to further enhance cosmetic outcome.

## Conclusion

The diagnostic and management were difficult and challenging not only because of the extremely rare disease, but also the multi-site involvement that require multidisciplinary approach. The disease put a very high burden as it requires a long hospital stay, numerous diagnostic evaluations, and also a long course of IV and oral antifungal therapy that is very expensive and on limited availability. Early surgical exenteration and debridement along with intravenous amphotericine B administration effectively controlled the infection.
